# Dengue Cases in the Eastern Region of Afghanistan, 2021–2023

**DOI:** 10.1007/s44197-025-00494-8

**Published:** 2025-11-29

**Authors:** Abdullah Jan Shinwari, Chatporn Kittitrakul, Mohammad Azim Azimee

**Affiliations:** 1 Department of Clinical Tropical Medicine, Faculty of Tropical Medicine, Mahidol University, Bangkok, Thailand; 2 Nangarhar Medical Faculty, Nangarhar University, Jalalabad, Afghanistan

**Keywords:** Dengue, Eastern Afghanistan, Epidemiology, Seasonal trends, Hospitalization

## Abstract

**Background and Aims:**

Dengue fever is an emerging public health concern in eastern Afghanistan, with reported cases increasing in recent years. This study investigated the epidemiologic distribution, demographic patterns, and clinical characteristics of dengue cases from 2021 to 2023 to identify temporal trends and guide control strategies in the region.

**Materials and Methods:**

A retrospective analysis of suspected and confirmed dengue cases from four eastern Afghan provinces (2021–2023) was conducted using HMIS data. WHO-defined suspected cases and laboratory-confirmed cases (NS1, IgM RDT/ELISA, RT-PCR) were included, with discordant results resolved by prioritizing RT-PCR and duplicates were removed. Demographic, clinical, hospitalization (>24 h), and outcome data were analyzed using descriptive statistics and logistic regression, with model fit assessed using Hosmer-Lemeshow.

**Results:**

Between 2021 and 2023, 3,766 dengue cases were reported in eastern Afghanistan, predominantly in Nangarhar (98.2%). Cases increased annually from 709 to 1,711, peaking in October–November. Most patients were male (63.9%) and aged 11–30 years (51.6%). Fever (98.2%), headache (96.2%), and myalgia (95.8%) were common; bleeding occurred in 2.6%. Hospitalization (admission for >24 hours) affected 382 patients (10.1%) and two deaths (0.05%) were recorded. Hospitalization was strongly associated with bleeding, province of residence, male gender, and infection during October–December (p < 0.05).

**Conclusion:**

Dengue cases in eastern Afghanistan, especially Nangarhar, increased 2021–2023, peaking late summer–autumn. Young adults and males were most affected, with bleeding predicting hospitalization. Strengthened vector control, community awareness, early diagnosis, and cross-border surveillance are critically important to preventing outbreaks.

## Introduction

Dengue virus infection is a major public health problem in tropical and subtropical regions, with nearly 50% of the global population living in dengue-endemic areas [1–4]. The annual global burden is estimated at 390 million infections, of which 96 million present with clinical manifestations [5]. The case fatality ratio (CFR) among untreated patients is approximately 10%, but it declines to 0.1% with appropriate management [6].

In recent years, large dengue outbreaks have occurred in neighboring countries of Afghanistan [6, 7]. In 2017, Khyber Pakhtunkhwa province of Pakistan reported 24,938 dengue cases, including 70 deaths (0.28%), with the highest incidence recorded in Peshawar District, which shares a border with Nangarhar Province in Afghanistan [8]. In 2019, the World Health Organization (WHO) documented the first confirmed dengue cases in Afghanistan, totaling 15 across six provinces—seven in Nangarhar, two in Paktia, one in Paktika, three in Kabul, and one each in Faryab and Laghman [9].

According to a recent WHO report, as of 2024, a total of 1,130 suspected dengue cases have been reported nationwide, with no associated deaths. Of these, 676 (59.8%) were females, and 10 (0.9%) were children under five years. Among 503 tested samples, 173 (34.4%) were confirmed positive by PCR. The number of suspected cases in 2024 exceeds the two-year average for 2021–2022 and surpasses the cases reported during the same period in 2023. In week 27 of 2024, 42 suspected cases were reported from Nangarhar Province, representing a 35.5% increase compared with the previous week [10].

Dengue cases are predominantly concentrated in eastern Afghanistan, largely due to close social and geographic connections with Khyber Pakhtunkhwa Province, Pakistan. This proximity significantly influences the health status of the population and facilitates cross-border transmission. Therefore, it is essential to examine the hospitalization patterns and epidemiologic distribution of dengue cases in this region. This study aimed to describe the epidemiologic distribution, demographic characteristics, and factors associated with hospitalization among dengue cases in eastern Afghanistan between 2021 and 2023.

## Materials and Methods

### Study Design and Setting

**Fig. 1:**
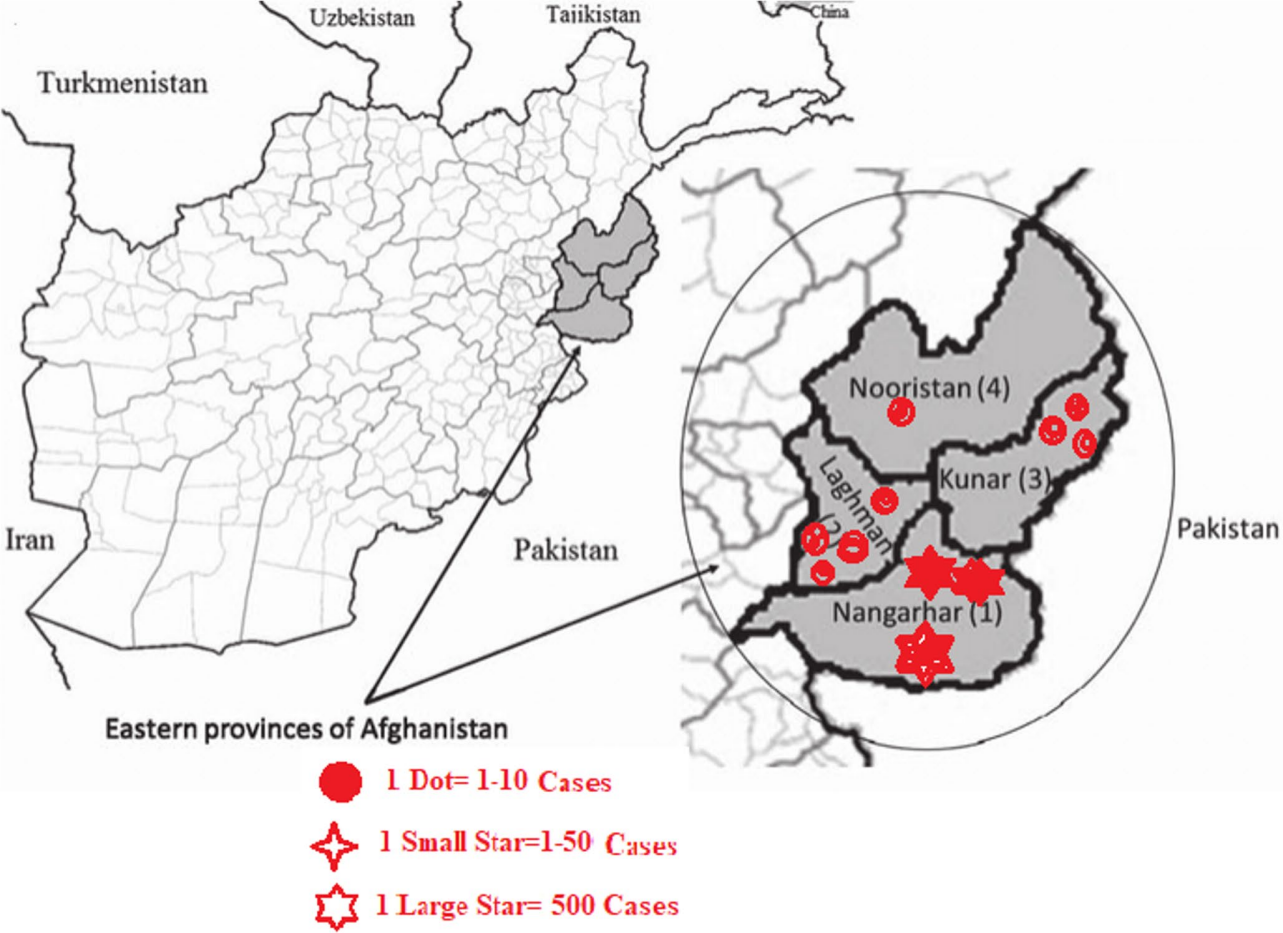
Geographic distribution of dengue cases in the eastern region of Afghanistan (Nangarhar, Kunar, Laghman, and Nuristan)

This retrospective descriptive study analyzed dengue cases reported from the eastern region of Afghanistan between January 2021 and December 2023. The study area included four provinces—Nangarhar, Kunar, Laghman, and Nuristan—where dengue transmission is most frequently observed. Data were obtained from over 260 public and private health facilities, ensuring broad coverage of the region. The eastern region of Afghanistan, bordering Khyber Pakhtunkhwa, Pakistan, is characterized by a hot semi-arid climate, rapid population growth, and substantial human mobility, all of which contribute to the risk of vector-borne disease transmission. The region serves as an important trade and transit corridor, influencing both the epidemiology and spread of dengue virus (DENV) infections (Fig. 1).

### Study Population and Sampling

The study included all laboratory-confirmed dengue cases reported from Nangarhar, Kunar, Laghman, and Nuristan provinces between January 2021 and December 2023, meeting the WHO dengue case definition. Data were extracted from the Malaria and Leishmania Information System (MLIS) and the National Disease Surveillance and Response (NDSR) system, both contributing to the national DHIS2 database under HMIS. Duplicate records were removed using unique identifiers, age, sex, and date of diagnosis.

Although HMIS collects both positive and negative results, only confirmed cases were retrieved, totaling 3,766 cases, each testing positive by NS1 antigen, IgM rapid test, IgM ELISA, or RT-PCR. Test-specific denominators or positivity rates could not be calculated.

Cases were aggregated at the provincial level for all four provinces, with district-level summaries provided only for Nangarhar, where case numbers were sufficient for meaningful analysis; district-level cases in Kunar, Laghman, and Nuristan were too few for detailed analysis. All datasets were validated, with district-level analyses limited to Nangarhar and descriptive summaries for the other provinces. This approach ensured an accurate and comprehensive depiction of dengue epidemiology despite variable data availability. Reporting and testing practices improved over 2021–2023, which may have influenced apparent increases in reported cases and laboratory positivity rates.

### Case Definition and Laboratory Testing

**Suspected Dengue Case**. A suspected dengue case was defined according to the World Health Organization (WHO) classification as an acute febrile illness accompanied by at least two of the following symptoms: headache, retro-orbital pain, myalgia, arthralgia, rash, or any hemorrhagic manifestation.

**Confirmed Dengue Case**. A confirmed dengue case was defined as a suspected case with laboratory confirmation using at least one diagnostic method, including NS1 antigen detection, dengue-specific IgM ELISA, rapid diagnostic test (RDT) for IgM, or reverse transcriptase polymerase chain reaction (RT-PCR) for dengue virus. Discordant test results were resolved by prioritizing the most sensitive and specific method (RT-PCR). Field surveillance teams also collected additional samples during outbreak investigations. Dengue virus serotyping was not performed due to limited laboratory capacity.

### Collection of Epidemiological, Socio-Demographic, and Clinical Data

For each reported case, epidemiological and clinical data were extracted from HMIS and surveillance reports, including age, sex, province, district, clinical manifestations (fever, headache, myalgia, bleeding), hospitalization, and outcome (recovery or death). Temporal data were categorized by year and season.

Potential surveillance and selection biases were considered in the study design, as case reporting may be higher in provinces with better health infrastructure (e.g., Nangarhar) and lower in remote provinces (e.g., Nuristan), which could influence hospitalization estimates.

The HMIS dataset does not include several key clinical indicators required for WHO 2009 dengue severity classification, such as abdominal pain, persistent vomiting, hepatomegaly, clinical fluid accumulation, hematocrit trends, shock, or organ involvement. Therefore, classification of cases into dengue without warning signs, dengue with warning signs, and severe dengue could not be performed.

### Data Quality Management

Data completeness and accuracy were ensured by cross-checking HMIS reports from over 260 health facilities across all four provinces. District-level validation was performed only for Nangarhar, where detailed district-level data were available; for Kunar, Laghman, and Nuristan, only provincial-level data could be verified. Outliers and duplicate entries were identified and removed based on unique identifiers, age, sex, and date of diagnosis. Only records meeting the WHO dengue case definition were included. Daily verification and resolution of inconsistencies were conducted in consultation with provincial health authorities to minimize potential surveillance and selection biases.

### Statistical Analysis

Data analysis was conducted using the Statistical Package for the Social Sciences (SPSS), version 26 (IBM Corp., Armonk, NY, USA). Descriptive statistics summarized demographic, temporal, geographic, and clinical characteristics. Categorical variables were reported as frequencies and percentages, while continuous variables were expressed as means ± standard deviations (SD).

Bivariable logistic regression was applied to examine associations between independent variables (age, sex, season, and clinical manifestations) and hospitalization. Variables with *p* < 0.25 in the bivariable analysis were included in the multivariable logistic regression model using stepwise backward elimination to adjust for potential confounders. Model goodness-of-fit was assessed using the Hosmer-Lemeshow test. Crude odds ratios (CORs) and adjusted odds ratios (AORs) with 95% confidence intervals (CIs) were reported. All statistical tests were two-tailed, with *p* < 0.05 considered statistically significant.

### Data Collection and Spatial Analysis

Detailed district-level dengue data were available only for Nangarhar; cases in Kunar, Laghman, and Nuristan were sparse and summarized at the provincial level. All datasets were thoroughly cleaned, cross-checked, and validated. District shapefiles for Nangarhar were unavailable, preventing spatial mapping. Consequently, district-level outputs are presented as Table 5 and narrative descriptions (Sect. 3.6).

## Result

### Overall Case Distribution

From January 2021 to December 2023, a total of 3,766 suspected and laboratory-confirmed dengue cases were reported across the eastern provinces of Afghanistan, including Nangarhar, Kunar, Laghman, and Nuristan. Among these, 382 patients (10.1%) required hospitalization, and two fatalities (0.05%) were documented, both due to dengue shock syndrome with severe hemorrhage.

The number of reported cases increased steadily over the three years—from 709 cases in 2021, 1,346 in 2022, and 1,711 in 2023—indicating a clear upward trend in dengue incidence (Table 1). This rise may partly reflect improvements in reporting practices, laboratory testing availability, and diagnostic strategies over time, in addition to actual increases in dengue transmission.

**Table 1:** Demographic characteristics of confirmed dengue cases by year and Province in the Eastern region of Afghanistan (2021–2023, *n* = 3,766)

Demographic data	2021, *n*, (%)	2022, *n*, (%)	2023, *n*, (%)	Total
Number of cases	709	1,346	1,711	3,766 (%)
Mean age, years (SD)	28.7 (14.7)	31.5 (15.0)	28.6 (16.8)	
Median age (years)	27	30	25	
Age group *n* (%)				
− 1–10 years	71 (10.0%)	82 (6.1%)	214 (12.5%)	367 (9.7)
− 11–20 years	173 (24.4%)	302(22.4%)	454 (26.5%)	929 (24.7%)
− 21–30 years	222 (31.3%)	398 (29.6%)	465 (27.2%)	1,085 (28.8%)
− 31–40 years	123 (17.3%)	257 (19.1%)	240 (14.0%)	620 (16.5%)
− 41–50 years	76 (10.7%)	177 (13.1%)	157 (9.2%)	410 (10.9%)
− 51–60 years	29 (4.1%)	96 (7.1%)	101 (6.0%)	226 (6.0%)
− 61–70 years	12 (1.7%)	24 (1.8%)	62 (3.6%)	98 (2.6%)
− 71–80 years	2 (0.3%)	8 (0.6%)	9 (0.5%)	19 (0.5%)
− 81–90 years		2 (0.1%)	9 (0.5%)	11 (0.3%)
Gender n (%)				
- Male	436 (61.5%)	1032 (76.7%)	938 (54.8%)	2,406 (63.9%)
- Female	273 (38.5%)	314 ((23.3%)	773 (45.2%)	1,360 (36.1%)
- Male: female ratio	1.6:1	3.7:1	1.2:1	1.8:1
Province n (%)				
- Nangarhar	709 (100%)	1341 (99.6%)	1649 (96.4%)	3,699 (98.1%)
- Nuristan	0	1 (0.07%)	2 (0.12%)	3 (0.08%)
- Kunar	0	1 (0.07%)	23 (1.34%)	24 (0.64%)
- Laghman	0	3 (0.22%)	37 (2.16%)	40 (1.06%)

### Demographic Characteristics

The mean age of patients ranged from 28.6 to 31.5 years, with a median age between 25 and 30 years. Most cases **(**51.6%**)** were aged 11–30 years, with the 21–30-year group forming the largest proportion across all years. A gradual increase was observed among children aged 1–10 years, rising from 10.0% in 2021 to 12.5% in 2023 (Table 1).

Males predominated each year, constituting 61.5% of cases in 2021, 76.7% in 2022, and 54.8% in 2023 **(**overall male-to-female ratio 1.8:1**)**, which may reflect both greater exposure due to occupational or social factors and potential reporting bias, as men are more likely to seek care.

Geographically, the majority of cases were reported from Nangarhar Province (98.2%), with smaller numbers from Laghman (1.06%), Kunar (0.64%), and Nuristan (0.08%), reflecting the distribution of dengue transmission in the eastern region.

### Clinical Characteristics and Laboratory Findings

All 3,766 patients were laboratory-confirmed, tested positive by at least one diagnostic method (NS1 antigen, IgM RDT, IgM ELISA, or RT-PCR).

Clinical symptoms were consistent across the three years (Table 2). The most frequently reported symptoms were fever (98.2%), headache (96.2%), and myalgia (95.8%). Nausea and vomiting increased over time, from 23.6% in 2021 to 60.4% in 2023, while bleeding remained uncommon but rose from 1.6% to 3.1% (Table 2).

**Table 2:** Clinical characteristics and outcomes of confirmed dengue cases in the Eastern region of Afghanistan (2021–2023, *n* = 3,766)

Variable	2021, *n*, (%)	2022, *n*, (%)	2023, *n*, (%)	Total
Symptoms (*n* (%))				
- Fever	646 (91.1)	1342 (99.7%)	1710 (99.9%)	3,698 (98.2%)
- Headache	613 (86.5%)	1321 (98.1%)	1687 (98.6%)	3,621 (96.2%)
- Myalgia	605 (85.3%)	1309 (97.3%)	1693 (98.9%)	3,607 (95.8%)
- Nausea/vomiting	167 (23.6%)	558 (48.9%)	1033 (60.4%)	1,758 (46.6%)
- Bleeding	11 (1.6%)	34 (2.5%)	53 (3.1%)	98 (2.6%)
Hospitalization	82 (11.6%)	106 (7.9%)	194 (11.3%)	382 (10.1%)
RDT IgM positive	132 (18.6%)	282 (20.9%)	527 (30.8%)	941 (25.0%)
NS1 positive	534 (75.3%)	1095 (81.3%)	1307 (76.4%)	2,936 (77.9%)
ELISA IgM positive	109 (15.5%)	220 (16.2%)	423 (24.7%)	752 (19.9%)
(%)	229 (32.3%)	531 (39.5%)	763 (44.6%)	1,523 (40.4%)
Outcome (n (%))				
- Recovery	709 (100%)	1345 (99.9%)	1710 (99.9%)	3,764 (99.9%)
- Death	0	1 (0.1%)	1 (0.1%)	2 (0.05%)

Hospitalization rates varied, peaking in 2021 (11.6%), decreasing to 7.9% in 2022, and rising again to 11.3% in 2023.

Laboratory results showed NS1 antigen positivity consistently above 75%, IgM RDT positivity rising from 18.6% to 30.8%, IgM ELISA positivity from 15.5% to 24.7%, and RT-PCR positivity from 32.3% to 44.6%, reflecting enhanced diagnostic capacity.

Patient outcomes were excellent, with recovery rates exceeding 99.9% and only two deaths (0.05%) (Table 2).

### Temporal and Seasonal Distribution

**Fig. 2:**
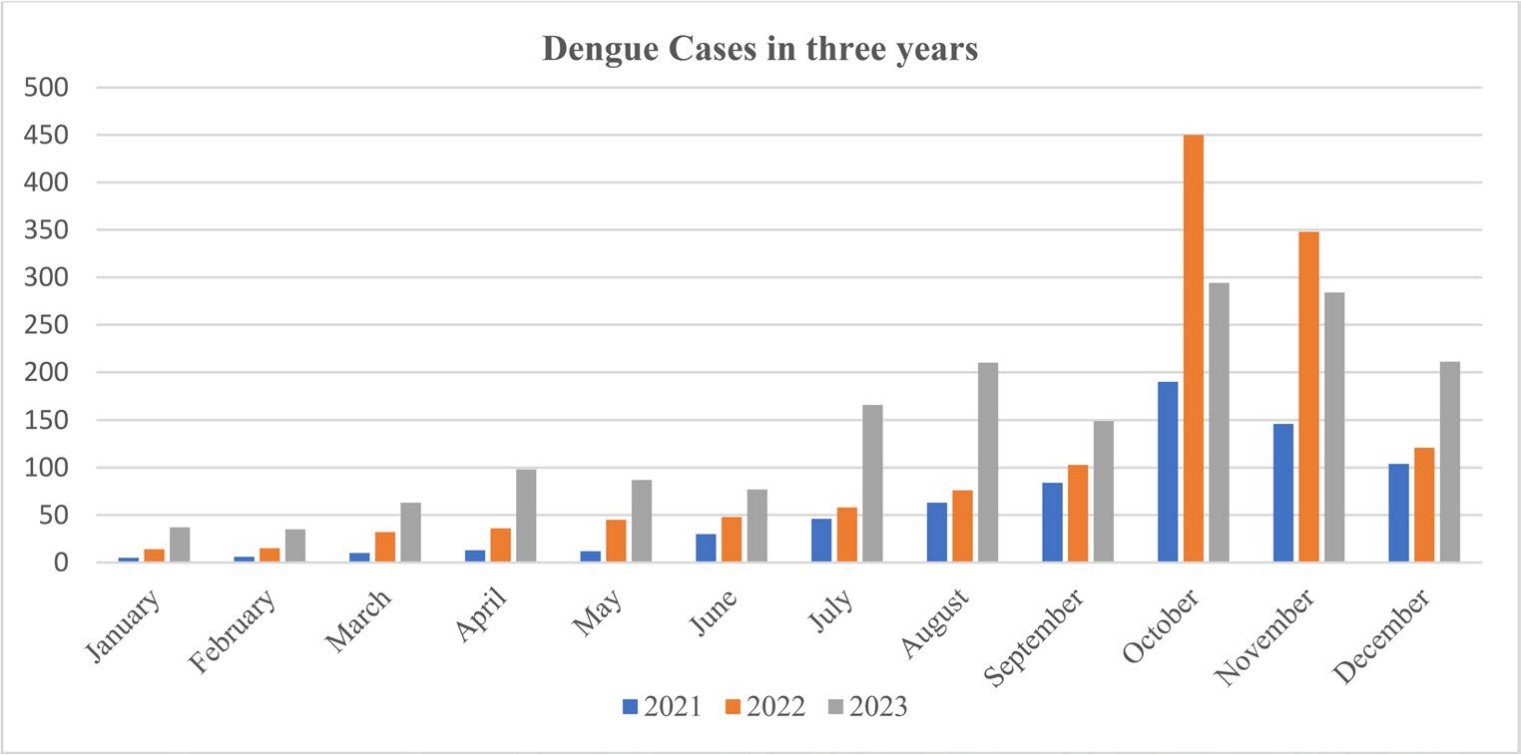
Monthly distribution of dengue cases in the Eastern Region of Afghanistan

Dengue cases in the eastern region of Afghanistan displayed a pronounced seasonal pattern, with peaks occurring between September and November, coinciding with the late summer–autumn months (post-monsoon period) and increased rainfall that favor Aedes mosquito breeding [11] (Fig. 2). In 2021, cases peaked in October (190; 26.8%), followed by November (147; 20.7%) and December (104; 14.7%). In 2022, the highest case numbers occurred in October (449; 33.4%) and November (348; 25.9%). In 2023, peaks were observed in October (293; 17.2%), November (283; 16.6%), and August (210; 12.3%), while transmission from January to May remained markedly low. Hospitalizations mirrored this seasonal trend, peaking between October and December, consistent with late summer–autumn months (post-monsoon period) rainfall and increased vector activity. Apparent elevated hospitalization proportions in winter months should be interpreted cautiously due to small absolute numbers and potential reporting variability. These temporal patterns should also be considered in the context of improved reporting and laboratory testing strategies during 2021–2023, which may have contributed to increases in reported cases and laboratory-confirmed positivity.

### Factors Associated with Hospitalization

Univariate and multivariable logistic regression analyses identified several factors significantly associated with hospitalization among dengue patients (Tables 3 and 4). Female patients were significantly less likely to be hospitalized compared with males (COR = 0.69, 95% CI: 0.56–0.88; *p* = 0.002; AOR = 0.68, 95% CI: 0.52–0.89; *p* = 0.004). Cases occurring during the late summer–autumn period from October to December had higher odds of hospitalization than those reported between January and September (COR = 1.73, 95% CI: 1.38–2.18; *p* < 0.001; AOR = 1.83, 95% CI: 1.41–2.37; *p* < 0.001). Province of residence was also a significant determinant: using Nangarhar as the reference, patients from Kunar (AOR = 8.08, 95% CI: 4.21–15.52; *p* < 0.001), Laghman (AOR = 2.91, 95% CI: 1.41–6.02; *p* = 0.004), and Nuristan (AOR = 6.10, 95% CI: 1.03–36.02; *p* = 0.046) exhibited higher odds of hospitalization, reflecting either increased disease severity or greater clinical caution in provinces with fewer cases. Bleeding manifestations emerged as a particularly strong predictor of hospitalization, with patients without bleeding showing markedly lower odds than those with bleeding (COR = 0.007, 95% CI: 0.003–0.014; *p* < 0.001; AOR = 0.007, 95% CI: 0.003–0.014; *p* < 0.001). Overall, these results indicate that gender, seasonal timing of infection, province of residence, and presence of bleeding are key determinants of hospitalization, though the wide confidence intervals for smaller provinces suggest caution in interpretation due to sparse data.

**Table 3:** Univariate analysis of epidemiological and clinical factors associated with hospitalization among confirmed dengue cases in the Eastern region of Afghanistan (*n* = 3,766)

Variable	Non-hospitalization, *n* = 3,384, (%)	Hospitalization, *n* = 382, (%)	Total, *n* = 3,766, (%)	COR (95% CI)	*p* value
Mean age (SD) (years)	29.63 (15.922) years	28.8 (15.196) years			
Median age (years)	27years	26years			
Age group (n (%))					0.368
− 1–20 years	1154 (88.8%)	146 (11.2%)	1300 (34.5%)	1.53(0.079–2.98)	
− 21–40 years	1537 (90.3%)	164 (9.7%)	1701 (45.2%)	1.29(0.66–2.51)	
− 41–60 years	572 (90.2%)	62 (9.8%)	634 (16.8%)	1.31(0.65–2.63)	
- >60 years	121 (92.4%)	10 (7.6%)	131 (3.5%)	1(reference)	
Gender (n (%))					0.002
- Male	1250 (88.8%)	111 (11.2%)	1361 (36.1%)	1(reference)	
- Female	2134 (91.8%)	271 (8.2%)	2405 (63.9%)	0.69(0.555–0.882)	
Season of Infection (Months Grouped) (*n* (%))					< 0.001
- January-September	1498 (92.6%)	120 (7.4%)	1618 (43.0%)	1(reference)	
** -** October-December	1886 (87.8%)	262 (12.2%)	2148 (57%)	1.73(1.383–2.175)	
Province (n (%))					< 0.001
- Kunar	28 (63.1%)	18 (36.9%)	46 (1.2%)	6.136(3.359-3.359. 11.207)	
- Laghman	41 (74.1%)	14 (25.9%)	55 (1.5%)	3.259(1.759–6.039)	
- Nuristan	3 (50.0%)	3 (50.0%)	6 (0.2%)	9.545(1.919-0.919. 47.470)	
- Nangarhar	3312 (90.5%)	347 (9.5%)	3659 (97.1%)	1(reference)	
Bleeding (n (%))					< 0.001
No	3377 (92.1%)	291 (7.9%)	3668 (97.4%)	1(reference)	
Yes	7 (7.1%)	91 (92.9%)	98 (2.6%)	0.007(0.003–014.003)

**Table 4:** Univariable and multivariable logistic regression analyses of epidemiological and clinical factors associated with hospitalization among conformed dengue cases in the Eastern region of Afghanistan (*n* = 3,766)

Variable	Crude OR (95% CI)	*p*-value		Adjusted OR (95% CI)	*p*-value
Gender (*n* (%))					
- Male	1(reference)			1(reference)	
- Female	0.69(0.555–0.882.555.882)	0.002		0.68(0.517–0.886)	0.004
Season of Infection (Months Grouped) (n (%))					
- January-September	1(reference)			1(reference)	
- October-December	1.73(1.383–2.175)	< 0.001		1.829 (1.410–2.374)	< 0.001
Province (n (%))					
- Kunar	6.136(3.359-3.359. 11.207)		< 0.001	8.080(4.206–15.522)	< 0.001
- Laghman	3.259(1.759–6.039)		< 0.001	2.908(1.406–6.016)	0.004
- Nuristan	9.545(1.919-0.919. 47.470)		0.006	6.100(1.033–36.018)	0.046
- Nangarhar	1(reference)			1(reference)	
Bleeding (n (%))					
Yes	1(reference)			1(reference)	
No	0.007(0.003–014.003)	< 0.001		0.007(0.003–014.003)	< 0.001

In the multivariable logistic regression analysis (Table 4), female patients had lower odds of hospitalization compared with males (AOR = 0.68, 95% CI: 0.52–0.89; *p* = 0.004), and cases occurring from October to December had higher odds than those from January to September (AOR = 1.83, 95% CI: 1.41–2.37; *p* < 0.001). Bleeding was a very strong predictor, with patients without bleeding showing markedly lower odds of hospitalization (AOR = 0.007, 95% CI: 0.003–0.014; *p* < 0.001), reflecting the low prevalence of hemorrhagic manifestations and near-perfect prediction of hospitalization in this subset.

Using Nangarhar as the reference, patients from Kunar (AOR = 8.08, 95% CI: 4.21–15.52; *p* < 0.001), Laghman (AOR = 2.91, 95% CI: 1.41–6.02; *p* = 0.004), and Nuristan (AOR = 6.10, 95% CI: 1.03–36.02; *p* = 0.046) had significantly higher odds of hospitalization, reflecting increased risk in provinces with fewer cases. Overall, gender, timing of infection, province of residence, and bleeding were significant determinants of hospitalization, though wide confidence intervals for smaller provinces indicate caution in interpretation due to sparse data (Table 4).

### Geographical Distribution of Dengue Cases in Nangarhar Province

Nangarhar Province accounted for the overwhelming majority of dengue cases in the eastern region of Afghanistan (3,699/3,766; 98.2%) (Table 5). To examine intra-provincial patterns, cases were disaggregated by district for 2021–2023. Jalalabad reported the highest cumulative number of cases (937; 19.4% of Nangarhar total), followed by Ghanikhil (693; 14.4%) and Mohmand Dara (644; 13.3%). Districts with very few cases included Kaga (3; 0.06%) and Dar-e-Nur (7; 0.2%), likely reflecting either low transmission or potential underreporting. Temporal trends varied across districts: Jalalabad experienced a consistent increase, rising from 46 cases in 2021 to 643 in 2023, whereas Mohmand Dara peaked in 2021 (411 cases) but declined sharply in subsequent years. These findings highlight substantial spatial heterogeneity within Nangarhar and underscore the need for targeted public health interventions in high-burden districts, providing a baseline for future spatial and temporal dengue surveillance.

**Table 5:** District-level distribution of confirmed dengue cases in Nangarhar Province by year (2021–2023, *n* = 3,699)

District	2021 (Freq, %)	2022 (Freq, %)	2023 (Freq, %)	Total (Freq, %)
Jalalabad	46 (6.2%)	248 (18.5%)	643 (38.9%)	937 (19.4%)
Ghanikhil	60 (8.1%)	399 (29.7%)	234 (14.2%)	693 (14.4%)
Mohmand Dara	411 (55.7%)	198 (14.7%)	35 (2.1%)	644 (13.3%)
Behsud	8 (1.1%)	155 (11.6%)	130 (7.9%)	293 (6.3%)
Batikot	8 (1.1%)	25 (1.9%)	211 (12.8%)	244 (5.0%)
Door Baba	137 (18.6%)	25 (1.9%)	14 (0.8%)	176 (3.8%)
Achin	14 (1.9%)	88 (6.5%)	60 (3.6%)	162 (3.3%)
Sorkhrod	7 (0.9%)	19 (1.4%)	87 (5.3%)	113 (2.3%)
Nazian	1 (0.1%)	65 (4.8%)	23 (1.4%)	89 (1.8%)
Rodat	0 (0.0%)	8 (0.6%)	23 (1.4%)	31 (0.6%)
Khogyani	1 (0.1%)	10 (0.7%)	19 (1.2%)	30 (0.6%)
Kama	1 (0.1%)	10 (0.7%)	18 (1.1%)	29 (0.6%)
Spinghar	0 (0.0%)	4 (0.3%)	18 (1.1%)	22 (0.5%)
Kot	1 (0.1%)	6 (0.4%)	14 (0.8%)	21 (0.4%)
Lalpora	4 (0.5%)	6 (0.4%)	11 (0.7%)	21 (0.4%)
Hisarak	2 (0.3%)	6 (0.4%)	11 (0.7%)	19 (0.4%)
Goshta	2 (0.3%)	7 (0.5%)	8 (0.5%)	17 (0.4%)
Khiwa	2 (0.3%)	4 (0.3%)	11 (0.7%)	17 (0.4%)
Agam	1 (0.1%)	4 (0.3%)	10 (0.6%)	15 (0.3%)
Shirzad	0 (0.0%)	0 (0.0%)	14 (0.8%)	14 (0.3%)
Haska Mina	0 (0.0%)	5 (0.4%)	6 (0.4%)	11 (0.2%)
Koz Kunar	0 (0.0%)	0 (0.0%)	10 (0.6%)	10 (0.2%)
Dar-e-Nur	0 (0.0%)	0 (0.0%)	7 (0.4%)	7 (0.2%)
Kaga	0 (0.0%)	0 (0.0%)	3 (0.2%)	3 (0.06%)
Total	709 (19.2%)	1341 (36.3%)	1649 (44.5%)	3,699 (100%)

## Discussion

This study provides an updated epidemiological overview of dengue cases in the eastern region of Afghanistan between 2021 and 2023, revealing a steady increase in reported infections. A total of 3,766 suspected and laboratory-confirmed cases were reported across Nangarhar, Kunar, Laghman, and Nuristan provinces, with 382 patients (10.1%) requiring hospitalization and two fatalities (0.05%) due to dengue shock syndrome with severe hemorrhage. The number of cases rose from 709 in 2021 to 1,711 in 2023, with the majority occurring in Nangarhar Province (98.1%), indicating that this province remains the epicenter of dengue transmission in the country. The rising trend is likely influenced by climatic and environmental conditions favorable to Aedes mosquito breeding, including high temperatures, humidity, and late summer–autumn (post-monsoon) water accumulation, as well as cross-border movement with dengue-endemic regions of Pakistan [6–10, 12, 13]. In contrast, provinces with cooler, more arid climates exhibited markedly lower dengue incidence. These observed trends may also be affected by differences in reporting and testing practices across provinces and years, which could contribute to apparent increases in case numbers and laboratory positivity.

Demographically, males consistently represented the majority of cases (63.9% overall), with a male-to-female ratio of 1.8:1, consistent with regional patterns reported in South and Southeast Asia [14–19]. This gender disparity likely reflects behavioral and occupational differences, as men are more frequently engaged in outdoor labor and less protected by clothing, while women’s indoor lifestyle and conservative dress may reduce exposure to Aedes bites. The age distribution showed that individuals aged 11–30 years accounted for over half of the cases, reflecting increased mobility, social interactions, and outdoor activity among this group [17–19]. A modest increase in cases among children aged 1–10 years was also observed over the study period.

Seasonal variation played a key role in dengue transmission patterns. The highest incidence was observed during autumn (September–November), following the monsoon season. This post-monsoon rise corresponds with increased mosquito breeding due to stagnant water accumulation and warm ambient temperatures that support mosquito survival and virus replication. Similar seasonal peaks have been reported in Pakistan, Bangladesh, and other tropical regions [16–18]. Agricultural harvesting activities during this time may also enhance human exposure to infected vectors.

Clinically, fever, headache, and myalgia were the predominant symptoms, observed in nearly all patients, while nausea, vomiting, and bleeding manifestations were less common but demonstrated an increasing trend over the three-year period. Comparable symptom patterns have been described in regional and global studies [19–21], underscoring the consistency of dengue’s core clinical presentation while highlighting variability in secondary symptoms influenced by local epidemiologic and host factors.

Logistic regression analysis identified several significant factors associated with hospitalization among dengue patients in the eastern region of Afghanistan. Female patients were significantly less likely to be hospitalized compared with males (COR = 0.69, 95% CI: 0.56–0.88, *p* = 0.002; AOR = 0.68, 95% CI: 0.52–0.89, *p* = 0.004), suggesting potential gender differences in disease severity or health-seeking behavior. Patients infected during the late-year period (October–December) had higher odds of hospitalization than those infected from January to September (COR = 1.73, 95% CI: 1.38–2.18, *p* < 0.001; AOR = 1.83, 95% CI: 1.41–2.37, *p* < 0.001), reflecting the association between seasonal timing and increased hospital care needs. Geographic location was also a determinant; patients residing in Nangarhar Province were more likely to require hospitalization compared with those in Nuristan (AOR = 0.12, 95% CI: 0.02–0.69, *p* = 0.018), while residence in Kunar and Laghman provinces did not show statistically significant differences. Importantly, bleeding manifestations emerged as the strongest predictor of hospitalization, with patients without bleeding having markedly lower odds of admission (COR = 0.007, 95% CI: 0.003–0.014, *p* < 0.001; AOR = 0.007, 95% CI: 0.003–0.014, *p* < 0.001), consistent with evidence linking hemorrhagic symptoms to severe dengue and increased need for inpatient care [22, 23]. Overall, these results indicate that gender, timing of infection, provincial residence, and bleeding manifestations are key determinants of hospitalization among dengue patients in this region.

Overall, these findings demonstrate that gender, seasonality, geography, and clinical severity are key determinants of dengue epidemiology and hospitalization risk in eastern Afghanistan. The pattern mirrors dengue behavior in neighboring endemic regions but underscores the country’s growing vulnerability to vector-borne diseases outbreaks.

### Conclusion

This study documents a clear upward trend in dengue cases in the eastern region of Afghanistan from 2021 to 2023, with Nangarhar Province being the most affected area. The post-monsoon season (September–November) was identified as the peak transmission period, driven by favorable environmental conditions for mosquito breeding. Males and young adults (11–30 years) were disproportionately affected, likely due to occupational and behavioral exposures. Fever, headache, and myalgia were the predominant symptoms, while bleeding manifestations were strongly associated with hospitalization and disease severity.

These findings underscore the urgent need for targeted vector control strategies, particularly before and during the monsoon season, and strengthened cross-border surveillance with neighboring dengue-endemic regions. Public health programs should also promote community awareness, environmental management, and early diagnosis and treatment to mitigate the rising dengue burden. Sustained epidemiologic monitoring and climatic risk assessment are essential to guide timely interventions and prevent future outbreaks in Afghanistan’s vulnerable eastern provinces. Future efforts should additionally focus on strengthening integrated regional surveillance systems, fostering cross-border cooperation, and enhancing real-time data sharing to enable timely detection and response to dengue outbreaks in the region.

### Strengths and Limitations

This study provides a comprehensive assessment of dengue epidemiology in eastern Afghanistan, analyzing 3,766 laboratory-confirmed cases reported over a three-year period. The use of a large, region-wide dataset from more than 260 health facilities enhances the representativeness of the findings and enables a detailed examination of temporal trends, demographic patterns, clinical characteristics, and hospitalization risk factors. The application of multivariable logistic regression further strengthens the robustness of the analytical approach.

However, this retrospective study is limited by reliance on routine surveillance data, which may have resulted in underreporting and incomplete clinical information. Dengue serotyping and WHO severity classification were not feasible, and key confounders—such as comorbidities, socioeconomic status, and environmental factors—were inconsistently available. Variability in data quality across facilities and the absence of official Nangarhar district shapefiles precluded spatial mapping, limiting district-level analyses to descriptive and tabular presentations.

## Data Availability

The data that support the findings of this study are available on request from the corresponding author. The data are not publicly available due to privacy and ethical restrictions .
